# Postoperative delirium does not affect cardiopulmonary exercise testing in aged patients undergoing cardiac valve surgery

**DOI:** 10.1186/s40981-022-00553-0

**Published:** 2022-08-13

**Authors:** Yuta Shimomoto, Kana Mukaiyama, Takashi Hori, Yuichirou Inaki, Takafumi Masai, Yukio Hayashi

**Affiliations:** 1 Rehabilitation and Cardiovascular Surgery, Sakurabashi-Watanabe Hospital, 2-4-32 Umeda, Kita-ku, Osaka, 530-0001 Japan; 2 Yoka Municipal Hospital, 1878-1, Yoka, Yoka-cho, Yabu, Hyougo 667-8555 Japan

**Keywords:** Cardiac rehabilitation, Postoperative delirium, Cardiopulmonary exercise testing, Heart surgery

## Abstract

**Background:**

The effect of delirium on cardiopulmonary exercise testing (CPX) is unknown. This retrospective study was to examine the effect of delirium on CPX at discharge in aged patients undergoing cardiac surgery.

**Methods:**

This study included seventy patients aged 70 or older undergoing cardiac valve surgery, who entered our ICU and were discharged from our hospital between June 2016 and July 2018. All patients received active exercise by our rehabilitation team from the first postoperative day and were performed a CPX on a cycle ergometer before discharge. The anaerobic threshold oxygen uptake and the slope of the relationship between carbon dioxide output and minute ventilation were examined. We obtained the patient’s data, including patient’s characteristics, cardiac function, anesthesia data, laboratory data, ICU data, and length of ICU and hospital stay. Data were analyzed by unpaired *t* test or Fisher’s exact test. *P* < 0.05 was considered statistically significant.

**Results:**

Of the 70 patients, 21 patients experienced delirium during ICU stay. The delirium group needed longer administration of sedatives and longer ICU stay and showed higher CRP value and lower renal function but similar cardiopulmonary function before discharge from our hospital compared with the non-delirium group.

**Conclusions:**

Patients with a history of delirium during ICU showed higher CRP value and lower renal function before discharge, but the CPX values at discharge were not significantly affected by delirium.

## Introduction

Delirium is an important predictor of negative clinical outcomes such as increasing mortality and cognitive impairment that negatively affect the quality of life in aged ICU patients [1]. Since early mobilization of ICU patients was recommended to reduce delirium in the clinical guideline of ICU patients [2], our rehabilitation team has introduced active exercise to the patients while they are receiving invasive ventilation.

Cardiopulmonary exercise testing (CPX) is useful in predicting the long-term mortality of patients undergoing surgery [3]; however, the effect of delirium on CPX is unknown. We also routinely perform CPX in ICU patients who underwent cardiac surgery before their discharge. This retrospective study was designed to examine the effect of delirium on CPX at discharge in aged patients who underwent cardiac valve surgery.

## Methods

This retrospective study was approved by the IRB of our hospital (Sakurabashi-Watanabe Hospital IRB ID: 18–92, approval date: 12/25/2018) and registered in the UMIN Clinical Trial Registry (UMIN 000,047,693), and informed consent was waived because of a retrospective design.

We included eighty-three patients aged 70 or older who underwent cardiac valve surgery under cardiopulmonary bypass, entered our ICU, and were discharged from our hospital between June 2016 and July 2018. All patients received active exercise from our rehabilitation team since the first postoperative day while on mechanical ventilation and performed a CPX on a cycle ergometer before discharge.

We obtained the following data from electrical medical records: patient’s characteristics, cardiac function before and after the operation, anesthesia data, laboratory data during the postoperative period, time of the first walk to the restroom, duration of rehabilitation, duration of sedative and catecholamine administration in ICU, and length of ICU and hospital stay. Cardiac function that we examined routinely in our hospital included left ventricular diastolic dimension (LVDd), left ventricular systolic dimension (LVDs), left ventricular ejection fraction (LVEF), early filling peak velocity (*E*), and early diastolic velocity of the mitral annulus by tissue Doppler imaging (*e*’).

An incremental symptom-limited exercise test was performed using an upright, electromagnetically braked cycle ergometer (Combi Wellness Aerobike 75 XL III®, Konami, Tokyo, Japan) before discharge from our hospital. Breath-by-breath oxygen uptake (VO_2_), carbon dioxide output (VCO_2_), and minute ventilation (VE) were measured throughout the test using an AE-310 s respiromonitor (Minato Medical Science, Osaka, Japan). Exercise began with a 4-min warm-up at 10 W, and the work rate was increased continuously at a rate of 10 w per minute (ramp protocol). The test was terminated when we obtained anaerobic threshold oxygen uptake (AT-VO_2_). In this study, we also obtained the slope of the relationship between VCO_2_ and VE. ΔVE/ΔVCO_2_ was calculated from start to end of incremental exercise by the least-squares linear regression.

In this study, we reviewed all electronic medical records of patients included and defined as delirium if one of the following symptoms was recorded in each patient’s medical record: disorientation; hallucination; self-withdrawal of drain tubes, intravenous lines, and endotracheal tubes; verbal abuse; violence; and sleep/wake cycle disturbances.

Our original hypothesis was that CPX data in patients with delirium were lower than those in patients without delirium. Since we did not find any previous data that was helpful for our study plan, we collected patients’ data for about 2 years (June 2016 to July 2018), planned to perform power of analysis, and calculated the number of additional patients to satisfy our hypothesis. However, as shown in the “Results” section, we did not obtain a clear difference in CPX data between the groups. Thus, we decided to cancel our original hypothesis. And then, we gave up the power of analysis, and we decided to report the data with the 83 patients collected for the 2 years.

### Statistical analysis

Data were expressed as mean ± SD. The data were analyzed by unpaired *t* test or Fisher’s exact test as appropriate. *P* < 0.05 was considered statistically significant.

## Results

**Fig. 1:**
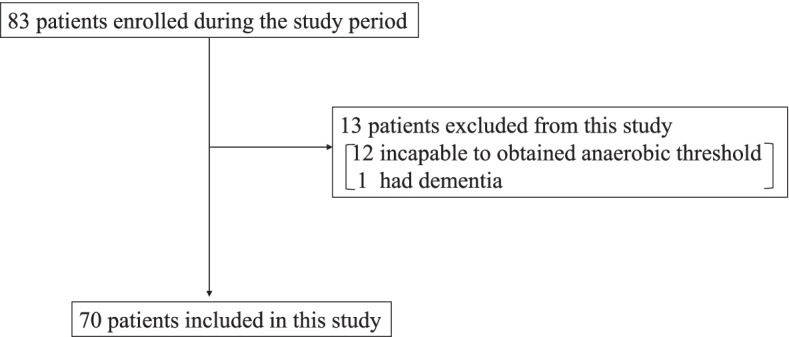
Patient inclusions and exclusions

**Fig. 2:**
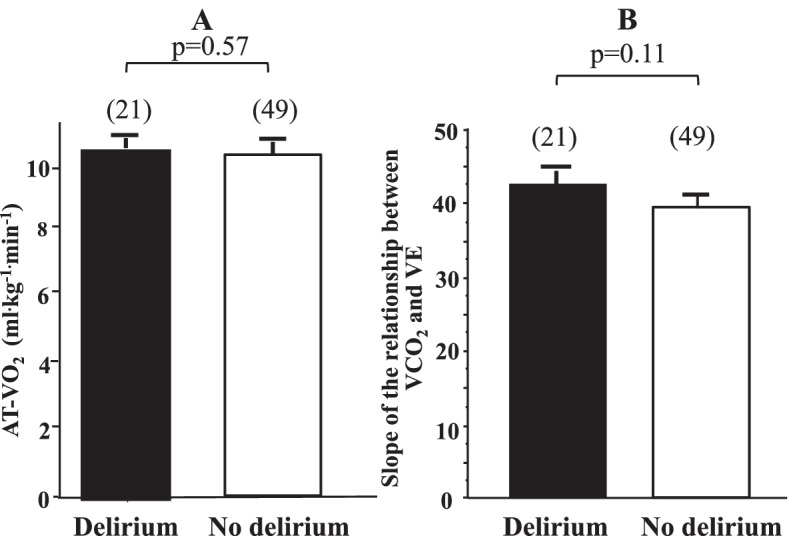
Anaerobic threshold oxygen uptake (AT-VO2) (**A**) and the slope of the relationship between carbon dioxide output (VCO2) and minute ventilation (VE) (**B**) in the presence and absence of delirium. Data are expressed as mean ± SD, and the number of observations is shown in parentheses

Although we recruited 83 patients in this study, 13 patients were excluded (Fig. 1). Thus, seventy patients were included in this study, of which 21 (30%) experienced delirium after ICU administration. Patients’ characteristics, preoperative conditions, and intraoperative data were similar between those with and without delirium (Table 1). Table 2 shows the summary of the postoperative data. The duration of sedative administration in ICU in the delirium group was significantly longer than that in the no delirium group, although the duration of intubation and duration of catecholamine administration were comparable between the two groups. The length of ICU stay in the delirium group was significantly longer than that in the no delirium group, while the length of hospital stay was comparable (Table 2). Table 3 presents cardiac function and laboratory data before discharge from our hospital. Cardiac function was comparable between the two groups. However, serum CRP and creatinine values in the delirium group were higher than those in the no delirium group, and the estimated glomerular filtration rate was significantly lower in the delirium group (Table 3). Figure 2 shows AT-VO_2_ (A) and the slope of VCO_2_ and VE (B) in the presence and absence of delirium, and both values were comparable between the groups.

**Table 1:** Preoperative and perioperative data in each group

	Delirium	No delirium	*P* value
*N*	21	49	
**Patient characteristics**			
Sex (male/female)	15/6	30/19	0.31
Age (years)	76 ± 3	75 ± 4	0.08
Height (cm)	160 ± 8	160 ± 9	0.96
Weight (kg)	61 ± 11	60 ± 11	0.65
**Preoperative cardiac function**			
LVDd (mm)	52 ± 9	48 ± 7	0.10
LVDs (mm)	34 ± 9	31 ± 7	0.07
LVEF (%)	63 ± 14	66 ± 10	0.28
*E*/*e*’	15.6 ± 10.3	15.6 ± 9.7	0.84
**Anesthesia data**			
Anesthesia time (min)	415 ± 109	383 ± 100	0.24
CPB time (min)	195 ± 79	164 ± 59	0.74
Water balance (ml)	367 ± 1587	920 ± 1087	0.10

*LVDd* Left ventricular diastolic dimension, *LVDs* Left ventricular systolic dimension, *LVEF* Left ventricular ejection fraction, *E* Early filling peak velocity, *e’* early diastolic velocity of the mitral annulus by tissue Doppler imaging, *CPB* Cardiopulmonary bypass. Data are expressed as mean ± SD

**Table 2:** Postoperative data in each group

	Delirium	No delirium	*P* value
*N*	21	49	
Duration of intubation (min)	1253 ± 861	997 ± 444	0.11
Duration of sedative administration (days)	3.0 ± 2.0	1.7 ± 0.7	< 0.01
Duration of catecholamine administration (days)	3.9 ± 2.0	3.4 ± 2.0	0.44
Time to first walk to a restroom (days)	4.8 ± 2.0	4.1 ± 1.6	0.15
ICU length of stay (days)	4.3 ± 2.1	3.1 ± 1.3	< 0.01
Hospital length of stay (days)	33 ± 11	33 ± 8	0.95

Data are expressed as mean ± SD

**Table 3:** Data before discharge from our hospital in each group

	Delirium	No delirium	*P* value
*N*	21	49	
Cardiac function before discharge			
LVDd (mm)	46 ± 7	45 ± 5	0.38
LVDs (mm)	33 ± 9	30 ± 6	0.14
LVEF (%)	59 ± 12	63 ± 10	0.28
*E*/*e*’	17.3 ± 8.7	17.8 ± 8.1	0.82
Laboratory data before discharge			
Hb (mg/dl)	10.1 ± 3.3	10.4 ± 2.0	0.63
Albumin (g/dl)	3.7 ± 1.0	3.5 ± 0.52	0.33
CRP (mg/dl)	6.7 ± 14.4	1.6 ± 6.1	0.04
eGFR (ml/min/1.73 m^2^)	50 ± 20	61 ± 16	0.01
Cr (mg/dl)	1.2 ± 0.7	0.9 ± 0.3	0.04

## Discussion

The principal finding of this study is that the CPX values including AT-VO_2_ and the slope of VCO_2_ and VE at discharge were not significantly affected by delirium in aged patients undergoing cardiac valve surgery.

It is well documented that the development of delirium after surgery is an important predictor to increase mortality [1, 4, 5], and this has also been confirmed in aged patients [4, 6]. Accordingly, recently published clinical practice guidelines in adult ICU patients show that delirium is an independent predictor of mortality [2, 7]. Furthermore, postoperative delirium is shown to be a risk factor that worsens the quality of life, such as activities of daily living, of aged surgical patients [8–10]. So far, however, there have been no clinical reports that demonstrate whether delirium in ICU affects cardiopulmonary function in aged patients undergoing cardiac surgery. Olofsson et al. [11] reported that delirium causes a negative impact on the rehabilitation outcome in elderly orthopedic patients, and Uthamalingam et al. [12] documented that delirium facilitates adverse effect on cardiac function in aged patients with heart failure. Based on these previous reports, we supposed that postoperative patients with delirium may show lower cardiopulmonary function compared to those without delirium. However, unexpectedly, the cardiopulmonary function we examined in this study was comparable between the patients with and without delirium (Fig. 2). Although we have no clear idea to explain this discrepancy between the previous data and our results, we are thinking of the possibility that early introduction of rehabilitation program might contribute to prevent adverse effect on the cardiopulmonary function in patients with delirium.

There are several parameters available with CPX to predict cardiac events in patients with heart disease. Peak VO_2_ is well recognized as a strong predictor [13]. However, the maximal exercise test may be risky for cardiac patients. Thus, we performed a submaximal exercise test and measured AT-VO_2_ instead of peak VO_2_, because these two factors were previously reported to show a significant correlation [14, 15]. AT-VO2 is a condition to be transitioned energetically to an obligatory anaerobiosis and identified by the increase of the VCO2/VO2. On the other hand, the slope of VCO_2_ and VE corresponds to the increasing ventilation in response to CO2 production, so it reflects increased ventilatory drive, and it has been reported to be a prognostic parameter for patients with heart disease [3, 16]. Thus, these parameters we used may be reasonable to predict future cardiac events of the patients we studied.

One may claim that the definition of delirium in this study may be doubtful because of the retrospective study design. We have to acknowledge that the definition was not so strict compared with that of a prospective study. However, as shown in Tables 2 and 3, the duration of sedative administration in ICU in the delirium group was significantly longer and serum CRP before discharge in the delirium group was significantly higher than those values in the no delirium group [17]. We think that these data may reflect the clinical characteristics of delirium, and our grouping may be reasonable in spite of the retrospective design.

There are several limitations in this study. First, although the present study showed that delirium did not affect cardiopulmonary function at discharge, our data did not guarantee a long-term outcome. Some reports documented that delirium in ICU may be associated with cognitive dysfunction after discharge [18, 19], and it is generally accepted that delirium during ICU stay is a risk factor of a long-term patient’s outcome [5, 6, 8]. Thus, it might be important to examine the effect of delirium in ICU on CPX values after 1 or more years after cardiac surgery, even if we did not find any difference between patients with delirium and without delirium just before discharge. Second, we did not consider the severity of delirium. Because of the retrospective design, we did not obtain the data required to estimate the severity. So, we have to acknowledge the possibility that a severe degree of delirium may affect the CPX values. Third, we did not examine the CPX values before the operation. So, we could not guarantee that the CPX values in the two groups were comparable before the operation. However, preoperative examination of CPX is not clinically and ethically available in patients scheduled for cardiac operation. Fourth, the subjects of our study were a small population of patients undergoing valve surgery, and we included all types of valve disease and operation. Thus, for example, if we limited the subjects to patients with aortic valve stenosis, another conclusion might be led. Now, we are accumulating the number of subjects and would like to present the data in the future.

## Conclusions

We concluded that cardiopulmonary ability evaluated by CPX at discharge was not significantly affected by the history of delirium in aged ICU patients undergoing valve surgery.

## Data Availability

The data that support the findings of this study are available from the corresponding author on reasonable reason.

## Abbreviations

CPX: Cardiopulmonary exercise testing
LVDd: Left ventricular diastolic dimension
LVDs: Left ventricular systolic dimension
LVEF: Left ventricular ejection fraction
*E*: Early filling peak velocity
*e*’: Early diastolic velocity of the mitral annulus by tissue Doppler imaging
VO_2_: Oxygen uptake
VCO_2_: Carbon dioxide output
VE: Minute ventilation
AT-VO_2_: Anaerobic threshold oxygen uptake
CTR: Cardiothoracic ratio
